# Psychometric Properties of the Dimensional Yale Food Addiction Scale for Children 2.0 among Portuguese Adolescents

**DOI:** 10.3390/nu16142334

**Published:** 2024-07-19

**Authors:** Ana Matos, Sílvia Félix, Carol Coelho, Eva Conceição, Bárbara César Machado, Sónia Gonçalves

**Affiliations:** 1 Psychology Research Center (CIPsi), School of Psychology, University of Minho, 4710-057 Braga, Portugal; pg49382@alunos.uminho.pt (A.M.); silviafelix@psi.uminho.pt (S.F.); pg47097@alunos.uminho.pt (C.C.); 2 Center for Psychology, Faculty of Psychology and Education Sciences, University of Porto, 4200-135 Porto, Portugal; econceicao@fpce.up.pt; 3 Research Centre for Human Development (CEDH), Faculty of Education and Psychology, Universidade Católica Portuguesa, 4169-005 Porto, Portugal; bcmachado@ucp.pt

**Keywords:** food addiction, psychometric properties, adolescence, grazing, intuitive eating

## Abstract

The dimensional Yale Food Addiction Scale for Children 2.0 (dYFAS-C 2.0) was developed to provide a reliable psychometric measure for assessing food addiction in adolescents, in accordance with the updated addiction criteria proposed in the fifth edition of the Diagnostic and Statistical Manual (DSM-5). The present study aimed to evaluate the psychometric properties of the dYFAS-C 2.0 among Portuguese adolescents and pre-adolescents and to explore the relationship between food addiction and other eating behaviors such as grazing and intuitive eating. The participants were 131 Portuguese adolescents and pre-adolescents (53.4% female and 46.6% male) aged between 10 and 15 years (M_age_ = 11.8) and with a BMI between 11.3 and 35.3 (M_BMI z-score_ = 0.42). Confirmatory Factor Analysis demonstrated an adequate fit for the original one-factor model (χ^2^ (104) = 182; *p* < 0.001; CFI = 0.97; TLI = 0.97; NFI = 0.94; SRMR = 0.101; RMSEA = 0.074; 95% CI [0.056; 0.091]). Food addiction was positively correlated with higher grazing (*r* = 0.69, *p* < 0.001) and negatively correlated with lower reliance on hunger/satiety cues (*r* = −0.22, *p* = 0.015). No significant association was found between food addiction and BMI *z*-score, or between food addiction and age. The results support the use of dYFAS-C 2.0 as a valid and reliable measure for assessing food addiction in Portuguese adolescents and pre-adolescents. Furthermore, the findings highlight that food addiction may be part of a spectrum of disordered eating behaviors associated with control impairment. Future research with a larger sample size could further elucidate the associations between food addiction and other variables, such as psychological distress and multi-impulsive spectrum behaviors.

## 1. Introduction

The globalization of food production systems centered on processed foods has led to a global increase in the consumption of energy-rich and nutrient-poor foods [1,2] This trend has negative consequences on weight maintenance, contributing to the development of overweight and obesity. Overweight and obesity are public health concerns because they impair physiological functions and increase the risk of developing other physical and psychological illnesses [1] For instance, hypertension, liver disease, insulin resistance, cardiovascular disease, diabetes, and psychosocial complications are often comorbid with overweight and obesity [1,3]. The prevalence of these conditions has nearly doubled since 1980, now affecting almost a third of the world’s population [1] Despite the known negative effects, the consumption of these foods continues to rise, possibly due to food addiction mechanisms [2,4].

The concept of food addiction has gained increasing research attention. Although its definition is still debated, a common understanding of food addiction has emerged by applying the criteria for substance dependence to eating behaviors. The available literature suggests that food addiction involves the excessive consumption of highly processed and hyperpalatable foods high in sugar, salt, and fat [2,5,6]. This consumption activates the brain reward system, releasing neurotransmitters like dopamine that increase pleasure and reinforcement, similar to substance dependence [5,6,7,8,9,10,11]. Some studies suggest that certain foods may have addictive potential and that repeated activation of the brain reward system can lead to physical and/or psychological dependence [6,9,11]. This pattern may be characterized by underlying mechanisms of loss of control over eating, binge eating behaviors, persistent desire to eat, and maladaptive efforts to reduce food consumption, increasing the risk of developing or maintaining overweight/obesity [9,12].

Adolescents may be particularly vulnerable to developing food addiction. The transition to adolescence is characterized by significant shifts in eating behavior that can persist into adulthood [13]. Understanding these behaviors, especially addictive ones, is crucial for early identification and prevention [14,15]. Biological, psychosocial, and cognitive changes during adolescence influence nutritional status and nutrient needs [16], while global rates of overweight and obesity among adolescents remain high [3]. Adolescents often snack, graze, skip meals, eat away from home, eat late at night, and consume fast foods [17]. Although food addiction can affect individuals at any age, adolescents are particularly susceptible due to their heightened neural and psychological vulnerability [14]. Specifically, adolescents often exhibit less effective emotional processing and cognitive control, and the neurobiological systems that regulate impulsivity are not fully mature [15,18]. This imbalance can lead to stronger reward-seeking behaviors and weaker inhibitory control [15,18], increasing the likelihood of engaging in risky behaviors such as substance use and poor eating habits [15,19].

The previous literature suggests that adolescents with food addiction exhibit more binge eating, depressive and anxious symptoms, impulsivity, and difficulties in executive functioning, which are related to difficulties in maintaining intuitive eating patterns (i.e., recognizing hunger and satiety cues) [15,20]. Other studies have shown that food addiction behaviors are positively associated with grazing eating behavior (i.e., grazing), characterized by repeated, unplanned ingestion of small amounts of food throughout a short period of time or during the day [8,21].

In this context, the Yale Food Addiction Scale [7] was developed to assess food addiction according to the Diagnostic and Statistical Manual for Mental Disorders-4 (DSM-4) [22]. With the development of the Diagnostic and Statistical Manual for Mental Disorders-5 (DSM-5) [23], Schiestl and Gearhardt (2018) [9] developed a dimensional version of the scale adapted for children and adolescents: the Dimensional Yale Food Addiction Scale for Children 2.0 (dYFAS-C 2.0). This instrument consists of 16 items evaluating seven of the criteria for Substance Use Disorders: loss of control, urge, inability to reduce behavior, abstinence, tolerance, continuous consumption despite the potential negative effects and time spent eating. The dYFAS-C 2.0 encompasses a dimensional and continuous representation of eating behaviors, rather than using categorical thresholds and clinical cutoffs [9,24,25].

Previous research has shown adequate psychometric properties for this measure in adolescents from the United States of America [9] and Denmark [24]. In Schiestl and Gearhardt (2018) [9], a one-factor structure model was partially supported, the instrument showed good internal consistency (α = 0.90) and food addiction was positively correlated with emotional eating, external eating, restrained eating and body mass index (BMI). Moreover, the food addiction scores did not differ between ages. In Horsager et al. (2021) [24], the one-factor model was supported, the instrument showed a higher internal consistency (α = 0.92), and food addiction was positively correlated with eating pathology, ADHD symptomatology, problematic use of alcohol, age, and BMI. Although the participants in this study had lower BMI than those of Schiestl and Gearhardt (2018) [9], a weaker correlation was found was found between food addiction and BMI.

To the best of our knowledge, no previous research has explored the use of dYFAS-C 2.0 with Portuguese adolescents or pre-adolescents. This gap hinders the examination of food addiction in this high-risk age group and the development of appropriate interventions for this problem. Thus, to better assess and understand adolescents’ eating behaviors, this study had two main goals: (1) to adapt the dYFAS-C 2.0 to Portuguese adolescents and pre-adolescents and analyze its psychometric properties; (2) to test associations between food addiction and grazing, intuitive eating, age, and BMI. We expect to fully support the one-factor model for the dYFAS-C 2.0 and that the instrument shows good internal consistency. We also hypothesize that food addiction will be associated with lower intuitive eating and higher grazing and BMI.

## 2. Methods

### 2.1. Participants

The inclusion criteria for this study were having Portuguese nationality and attending the fifth to ninth year of school. This sample consisted of 131 Portuguese adolescents and pre-adolescents, aged between 10 and 15 years (*M* = 11.8; *SD* = 1.36), with 70 (53.4%) female and 61 (46.6%) male participants. The participants were predominantly in the fifth (26%) and seventh (25.2%) grades. The mean BMI was 20.1 (*SD* = 3.97), with a *z*-score for age of 0.42 (*SD* = 1.10). Most of the participants (65.65%; *n* = 86) were between the >5th and <85th percentiles, indicating a healthy weight for their age. The remaining participants were underweight (2.29%; *n* = 3), overweight (13.74%; *n* = 18), or obese (10.69%; *n* = 14).

### 2.2. Measures

#### 2.2.1. Anthropometric Data

A SECA 899 flat scale model (SECA Corp., Hamburg, Germany) was used to collect weight. Height was assessed using a portable stadiometer.

#### 2.2.2. Sociodemographic Questionnaire

This questionnaire included information about age, sex, nationality, and year of schooling.

#### 2.2.3. The Dimensional Yale Food Addiction Scale for Children 2.0 (dYFAS-C 2.0)

The dYFAS-C 2.0 consists of 16 self-report items measured on a 5-point Likert scale, where 0 = never; 1 = rarely; 2 = sometimes; 3 = very often and 4 = always. Responses are reported based on the last 12 months. Scores are continuous and dimensional, ranging from 0 to 64, with higher scores indicating more severe food addiction behavior [9]. A Portuguese translation developed for this study was used. The internal consistency of this study was α = 0.87, and the response rate was 91.6%.

#### 2.2.4. Repetitive Eating Questionnaire (Rep(eat)-Q)

The Rep(eat)-Q evaluates grazing divided into two subtypes: compulsive grazing (CG), in which the individual cannot resist the temptation to eat, and repetitive eating (RE), associated with eating repeatedly in a distracted and irrational manner. The instrument consists of 12 self-reported items, answered on a 7-point ordinal scale, where 0 = never and 6 = more than once a day. Participants responded considering the frequency of grazing over the last 28 days. The total score can range from 0 to 90, with higher scores indicating more grazing [21,26]. The internal consistency of this study was α = 0.87, and the response rate was 100%.

#### 2.2.5. Intuitive Eating Scale-2 (IES-2)

The IES-2 evaluates an individual’s tendency to respond to physiological signals of hunger and satiety. The adolescent version contains three subscales: “Confidence in hunger and feeling of satiety (RHSC)”; “Eating for physical rather than emotional reasons (EPR)” and “body–food–choice congruence (BFCC)”. The scale consists of 23 items, evaluated on a 5-point Likert scale where 1 = strongly disagree and 5 = strongly agree. Higher total scores correspond to higher levels of intuitive eating, indicating positive eating habits [20,27]. The internal consistency for this study was α= 0.61, and the response rate was 93.2%.

### 2.3. Procedure

After receiving permission from the scale authors [9], the dYFAS-C 2.0 was translated by one author and then back-translated by another author. Discrepancies were discussed to reach a final version. Before recruitment and data collection, the scales were administered to three adolescents to ensure comprehension.

This study received approval from the Ethics Committee of the University of Minho (CEICSH 064/2019). Initial contact was made with several schools to publicize the study and recruit participants. After obtaining school approval, teachers provided informed consent forms to parents/guardians and students, ensuring voluntary, confidential, and anonymous participation. Parent consent and child assent were obtained. Only students with prior authorization participated in the study.

Data collection followed a cross-sectional design and took place in three public schools in the north of Portugal. The questionnaires were completed using paper-and-pencil format, with the order of presentation of the questionnaires randomized. After completing the questionnaires, the students were weighed and measured without shoes.

### 2.4. Data Analysis

Statistical analysis was conducted using IBM SPSS Statistics (Version 28.0) [28] and Jamovi (Version 2.4.8.0) [29]. Descriptive statistics and frequencies for categorical variables were calculated to characterize the participants according to sociodemographic and anthropometric data. Data normality was assessed considering severe deviations from univariate normality for absolute values of asymmetry |sk| > 3 and kurtosis |ku| > 10 [30].

Confirmatory Factor Analysis (CFA) was performed to analyze the underlying factorial structure of the dYFAS-C 2.0 and evaluate the fit of the model. The lavaan package was used and the weighted least squares method (Diagonally Weighted Least Squares–DWLS) was selected [31]. Fit indices included thecomparative fit index (CFI), normalized fit index (NFI), Tucker–Lewis index (TLI), goodness of fit index (GFI), Hoelter critical N (CN), standardized root mean square residual (SRMR), root mean square error of approximation (RMSEA), and adjustment index (χ^2^). A model is considered satisfactory when CFI, NFI, TLI, and GFI ≥ 0.90–0.95; CN ≥ 100; SRMR ≤ 0.08 and RMSEA ≤ 0.05 [32,33].

To determine the reliability of the internal consistency of dYFAS-C 2.0, ordinal Cronbach’s alpha (α) and ordinal McDonald’s omega (ω) were analyzed, with preferable values between 0.80 and 0.90 [34]. McDonald’s ω is estimated based on CFA, namely on factor loadings and error variances. Cronbach’s alpha (α) coefficients were calculated for the other instruments used in the present study (Rep(eat)-12 and IES-2), considering the same preferable values.

Pearson’s correlation coefficients were calculated for dYFAS-C 2.0, Rep(eat)-12, IES-2, BMI *z*-score, and age. A significance level of 5% (α = 0.05) was used for all the statistical analyses mentioned above.

## 3. Results

### 3.1. Food Addiction and Psychometric Properties of dYFAS-C 2.0

The descriptive statistics of the dYFAS-C 2.0 items are presented in Table 1. There were no severe violations of univariate normality, as the absolute values of asymmetry and kurtosis were both lower than 3 and 10, respectively.

Table 2 displays the CFA results, indicating an adequate fit for the original one-factor model [9]. Furthermore, dYFAS-C 2.0 demonstrated good internal consistency. Cronbach’s alpha was 0.87 and McDonald’s omega 0.87.

Factor loadings for the 16 items of the dYFAS-C 2.0 ranged from 0.238 (item 6) to 0.822 (item 15), all with *p* < 0.001, indicating a moderate to strong association between the observed variables and the latent variable (factor)—food addiction (see Table 3).

### 3.2. Validity Evidence Based on the Relationship between Food Addiction and Other Variables

Table 4 presents Pearson’s correlation coefficients for the association between the total score of dYFAS-C 2.0 and the remaining measures. The results show a positive correlation between the dYFAS-C 2.0 total score and the Rep(eat)-12 total score (*r* = 0.69, *p* < 0.001), as well as with its subscales compulsive grazing (*r* = 0.72, *p* < 0.001) and repetitive eating (*r* = 0.56, *p* < 0.001). In respect to the IES-2, there was only one significant correlation with the subscale reliance on hunger/satiety cues (*r* = −0.22, *p* = 0.015). No significant correlations were found between the dYFAS-C 2.0 total score and BMI z-scores or between the dYFAS-C 2.0 total score and age.

## 4. Discussion

The present study is the first to adapt a measure to assess food addiction (i.e., dYFAS-C 2.0) in Portuguese adolescents and to evaluate the psychometric properties of its translated version into Portuguese. Overall, the findings support that the dYFAS-C 2.0 presents adequate psychometric properties and good validity evidence in Portuguese adolescents based on internal structure, construct validity, reliability, and its relationship with other variables.

The results showed an adequate fit for the one-factor model proposed by Schiestl and Gearhardt (2018) [9], with all items retained. Factor loadings were consistent and comparable to those reported in previous studies [9,24], indicating the robustness of the dYFAS-C 2.0 items across different samples. Regarding the reliability of the instrument, the results revealed good internal consistency, indicating that the scale items are homogeneous and measure the same construct consistently.

Concerning the relation with other variables, positive associations were observed between the dYFAS-C 2.0 total score and both the total score and the subscales of Rep(eat)-Q, indicating a strong correlation between food addiction and grazing. This finding aligns with existing research [8,35,36], suggesting that compulsive grazing and repetitive eating behaviors reinforce tendencies toward food addiction. According to [8], compulsive grazing may emerge as an addictive behavior in response to food. It is important to note that, while food addiction and grazing represent distinct eating behaviors, both contribute to maladaptive and unhealthy patterns associated with excessive food consumption. Thus, it can be inferred that food addiction and grazing assess similar constructs within the spectrum of eating pathology characterized by impaired control. On the one hand, there is a prevalence of excessive consumption of highly processed and hyperpalatable foods during adolescence [14]. On the other hand, there are difficulties in resisting the urge to eat and/or the consumption of small amounts of food in a repetitive and unplanned manner [21]. Moving forward, potential shared pathways between food addiction and grazing should be explored in future studies to clarify adolescents’ eating behaviors and the factors that sustain these possible underlying difficulties in impulse control.

No significant relationship was found between the dYFAS-C 2.0 and the IES total scores. However, the dYFAS-C 2.0 total score had a negative correlation with the IES “reliance on hunger/satiety cues” subscale. Based on these results and considering those obtained by Tylka and Kroon Van Diest (2013) [26], we can speculate that adolescents who experience difficulties in the recognition of physiological cues of hunger and satiety may be more prone to guide their eating behaviors based on external cues, such as emotional changes or distress. If so, adolescents may be more predisposed to develop food addiction behaviors in which a pattern of excessive consumption of foods with high levels of sugar, salt, and fat may arise [5]. To the best of our knowledge, the present study is the first to investigate the association between food addiction and intuitive eating. Nevertheless, previous studies have found negative associations between intuitive eating and other maladaptive eating patterns such as binge eating and grazing [20,37], endorsing the divergence between these constructs.

While Schiestl and Gearhardt (2018) [9] and Horsager et al. (2021) [24] found a positive (although weak) correlation between the dYFAS-C 2.0 total score and the BMI *z*-score, the present study found no significant correlation between these variables. This may be attributed to the predominantly normal weight distribution among participants. Accordingly, although the percentages of overweight and obesity in our sample reflect the national trends found in Portuguese children and adolescents [38], only 24.43% of our participants presented overweight or obese. This rate is significantly lower than that reported in Schiestl and Gearhardt’s (2018) [9] study, where nearly half of the participants (48.8%) had overweight/obesity. Although this may be a surprising result, it also suggests that dYFAS-C 2.0 is able to capture food addiction behavior even in those with smaller BMIs, as suggested by the study by Horsager and colleagues (2021) [24].

Additionally, the dYFAS-C 2.0 scores were not correlated with age, which corroborates the results of Schiestl and Gearhardt (2018) [9], where no significant differences were found in the dYFAS-C 2.0 scores between participants aged 13 to 16 years. These results suggest that food addiction behaviors manifest similarly across different ages and body weights during adolescence.

### 4.1. Implications

Adolescents are particularly vulnerable to developing food addiction behaviors [14], and these changes in eating behavior can persist into adulthood [13]. From a clinical perspective, having a reliable tool to measure food addiction represents a significant advancement that enables early and effective assessments and intervention for adolescents with disordered eating. Additionally, dYFAS-C 2.0 can be used alongside other measures that assess maladaptive eating patterns, such as grazing or adaptive eating patterns like intuitive eating. Using the dYFAS-C 2.0 can also aid in identifying the risks and predispositions for developing health issues associated with food addiction. At the research level, the psychometric validation of this tool facilitates the expansion of research focused on food addiction among adolescents, a high-risk group for the onset of disordered eating behaviors. This contributes to improved assessment and the development of appropriate interventions. The dissemination and adaptation of the dYFAS-C 2.0 to other cultures, such as the Portuguese, enhances understanding of adolescents’ eating behaviors and enables cross-cultural comparisons regarding food addiction.

### 4.2. Limitations and Recommendations for Future Studies

The limitations of the present study include the small sample size and data collection confined to the north of Portugal, which restricts the generalizability of the findings. Future research would benefit from larger sample sizes to enhance the robustness of the results and should investigate the relationships between food addiction and other variables, such as psychological distress and multi-impulsive spectrum behaviors in adolescents. Moreover, it would be valuable to examine food addiction behaviors in clinical samples, particularly among adolescents with overweight and eating disorders. Understanding the association between food addiction, emotion regulation, and general well-being in clinical settings could provide insights into effective intervention strategies.

## 5. Conclusions

The Portuguese version of the dYFAS-C 2.0 demonstrated robust psychometric properties and proved to be a reliable and valid tool for assessing food addiction behaviors in adolescents and pre-adolescents. Food addiction was related to increased grazing and reduced reliance on hunger/satiety cues. Additionally, in our sample of adolescents, food addiction was not related to BMI or age.

## Figures and Tables

**Table 1:** Descriptive statistics and data normality: dYFAS_C 2.0.

Item	*M*	*SD*	Asymmetry	Kurtosis	Cronbach’s Alpha if Item Deleted
dYFAS_C_1	0.95	0.93	0.80	0.11	0.85
dYFAS_C_2	0.99	0.99	1.01	0.93	0.85
dYFAS_C_3	0.41	0.78	2.05	4.14	0.86
dYFAS_C_4	1.26	1.03	0.56	−0.14	0.86
dYFAS_C_5	0.52	0.84	1.82	3.61	0.86
dYFAS_C_6	0.82	1.01	1.40	1.66	0.87
dYFAS_C_7	0.41	0.89	2.47	5.82	0.86
dYFAS_C_8	0.32	0.67	2.64	8.48	0.86
dYFAS_C_9	0.77	1.00	1.57	2.20	0.86
dYFAS_C_10	0.34	0.71	2.57	7.49	0.87
dYFAS_C_11	0.64	0.91	1.67	3.02	0.86
dYFAS_C_12	0.80	0.87	1.06	1.30	0.86
dYFAS_C_13	1.18	1.16	0.73	−0.34	0.85
dYFAS_C_14	0.82	1.02	1.07	0.32	0.85
dYFAS_C_15	1.00	1.09	0.98	0.34	0.85
dYFAS_C_16	1.01	1.13	1.00	0.31	0.85
dYFAS_C_Total	11.8	8.45	1.13	2.07	-

**Table 2:** Fit indices.

	χ^2^	Df	CFI	TLI	NFI	GFI	CN	SRMR	RMSEA (95% CI)
dYFAS-C	182	104	0.97	0.97	0.94	0.97	104.87	0.101	0.074

Note: χ^2^—adjustment index; df—degrees of freedom; CFI—comparative fit index; TLI—Tucker–Lewis index; NFI—normalized fit index; GFI—goodness of fit index; CN—Hoelter critical N; SRMR—standardized root mean square residual; RMSEA—root mean square error approximation.

**Table 3:** Factor loadings of confirmatory factor analysis.

Latent Variable	Observed Variable	Factor Loadings	*p*
dYFAS-C 2.0	dYFAS_C_1	0.676	<0.001
	dYFAS_C_2	0.640	<0.001
	dYFAS_C_3	0.552	<0.001
	dYFAS_C_4	0.652	<0.001
	dYFAS_C_5	0.485	<0.001
	dYFAS_C_6	0.238	<0.001
	dYFAS_C_7	0.513	<0.001
	dYFAS_C_8	0.377	<0.001
	dYFAS_C_9	0.627	<0.001
	dYFAS_C_10	0.356	<0.001
	dYFAS_C_11	0.699	<0.001
	dYFAS_C_12	0.387	<0.001
	dYFAS_C_13	0.706	<0.001
	dYFAS_C_14	0.726	<0.001
	dYFAS_C_15	0.822	<0.001
	dYFAS_C_16	0.738	<0.001

**Table 4:** Correlation matrix.

Variable	1	2	3	4	5	6	7	8	9	10
1. dYFAS-C 2.0	-									
2. Rep(eat)-12	0.69 ***	-								
3. Rep(eat)_CG	0.72 ***	0.90 ***	-							
4. Rep(eat)_RE	0.56 **	0.93 ***	0.67 ***	-						
5. IES-2	−0.17	0.04	0.03	0.04	-					
6. IES_RHSC	−0.22 *	−0.07	−0.01	−0.10	0.76 ***	-				
7. IES_BFCC	−0.16	0.03	−0.02	0.03	0.60 ***	0.45 ***	-			
8. IES_EPR	0.02	0.07	0.07	0.07	0.65 ***	0.15	0.22 **	-		
9. BMI Z_Score	0.05	0.03	0.10	−0.04	−0.09	−0.16	−0.06	0.07	-	
10. Age	0.15	0.18 *	0.15	0.17 *	0.11	0.04	0.12	0.08	0.08	-

Note: dYFAS-C 2.0—total scores; Rep(eat)-12—total scores; Rep(eat)_CG—compulsive grazing subscale of the Rep(eat)-12; Rep(eat)_RE—repetitive eating subscale of the Rep(eat)-12; IES-2—total scores; IES_RHSC—reliance on hunger/satiety cues subscale of the IES-2; IES_BFCC—body–food–choice congruence subscale of the IES-2; IES_EPR—eating for physical reasons subscale of the IES-2. * *p* < 0.05, ** *p* < 0.01, *** *p* < 0.001.

## Data Availability

The data presented in this study are available on request from the corresponding author due to the data are part of an ongoing study.
